# Mr. Ring, on Vaccination

**Published:** 1806-06

**Authors:** John Ring

**Affiliations:** New Street, Hanover Square


### *To the Editors of the Medical and Physical Journal.*

GENTLEMEN,

IN the 19th Volume of the General Medical Journal, published at Paris, is an analysis of Dr. De Carro's History of Vaccination in Turkey, Greece, and the East Indies, of which I some time since gave an account in this publication. The analysis in the French Journal is by Dr. Valentin, who has added several notes; and among others, one respecting the best mode of preserving and conveying cow-pock matter. It is much to be regretted, that these hints are still necessary; and many serious inconveniencies arise from want of attention to this subject.

Dr. Valentin observes, that the ivory lancets of Dr. De Carro succeed at a great distance; and are at once the vehicle and the instrument. It is, however, always most adviseable, to make a puncture first with a common lancet. He says, he has seen them adopted in London, by the friends of Jenner. The instrument, however, which Dr. Valentin saw used for this purpose in London, by others as well as myself, was not exactly the same as that sent to me by Dr. De Carro; but a vaccinator, a mere ivory point, or lancet in miniature; such as I described in your Journal for April, 1803. These are, indeed, adopted very extensively; and, I trust, will be universally adopted; unless a better method can be pointed out.

It

It appeared to me, that a vaccinator would be an improvement of the ivory lancet; being a hundred times lighter, smaller, and cheaper than the lancet, when fixed in a handle, and secured in a case. This is of some consequence to those, who give them away in considerable numbers; or receive them by the post.

Dr. Valentin agrees in opinion with those, who condemn the practice of taking matter at a late period; and that of making more than one puncture in one arm.—When in England, he was not become a perfect convert to the opinion of Dr. Jenner, that the cow-pock derives its origin from the grease. He stated reasons for his scepticisms to Dr. Jenner; who urged several arguments in favour of his own opinion, particularly the conversion of Dr. Sacco; and referred him to me for further information. I shewed him many instances in my Treatise. His doubts, however, and those of the French physicians in general, could not be dispelled all at once.

He now tells us, " Mons. Godine, Professor of the Veterinary College at Alfort, has successfully inoculated different animals with the matter of grease; so that he also augments the number of those foreigners, who concur in justifying the theory of that great man, to whom all human nature pays homage."

Mons. Godine, as well as Dr. Valentin, has succeeded in inoculating sheep with vaccine matter from the human subject. Dr. Valentin has re-transmitted it through the human subject. Mons. Godine has not done this; but he has inoculated animals with vaccine matter taken from those of the same species; and thinks he has thus preserved them from the epidemic disorder to which they are naturally subject.

" It is, therefore, evident, says Dr. Valentin, " that the cow is not the only animal, susceptible of the infection of grease on its nipples; or capable of communicating it to other quadrupeds; of which the following fact, related to me, in June last, by Jenner, is a very remarkable instance. An ewe, having yeaned three lambs, of which two died, was troubled with a superabundance of milk; and a servant undertook to milk her, who was at the same time employed in the cure of a horse which had the grease; in consequence of which pustules appeared on her nipples, similar to those which we see on the nipples of cows. A veterinary surgeon declared, that these might be communicated nicated from the ewe to cows; but the farmer smiled, and did not believe it; nevertheless he consented, at the request of Mr. Henry Jenner, that the same servant should milk his cows immediately after the ewe. In consequence of this, the cows were infected; and they afterwards infected one of the dairy-maids."

Dr. Valentin observes, that during his stay in England, he had made particular enquiries concerning the sheep-pox, but without success; but that Dr. Jenner, and some of his friends, had promised him to pursue a farther investigation of the subject. The respect I entertain for Dr. Valentin, induced me to begin, and to continue my researches, till I had obtained the most full and satisfactory information on this point; which may be found in the Medical Journal for November, 1804.

I was lately told, that this disease had appeared in the neighbourhood of High Wycomb, and in that of Sittingborne; but these reports proved to be erroneous. The following letter from Mr. Murton of Tunstall, near Sittingborne, may perhaps furnish a valuable hint to some persons, who are occupied in rural affairs.

"I am sorry I cannot give you any satisfactory account of the disease, concerning which you have written to me. I have been in the habit of attending to sheep from my earliest years; and never knew, or heard, of their having any thing like the disorder you mention. I recollect perfectly well, that at the time when Mr. Dyne saw our late friend Mr. Woollett's flock, they were afflicted, not with the sheep-pox, but with the scab, or shab; which is considered to be similar to the mange in dogs, or the itch in the human subject. The cure consists of an outward application, of strong tobacco water and oil of turpentine."

As a supplement to Dr. Valentin's notes on De Carro's History of Vaccination in India, I shall here subjoin some interesting intelligence on the same subject; which I have derived from different sources of information. The first is an extract of a letter from Mr. S. D. White to Mr. Ford, dated Madras, March 4, 1805. It is as follows: "I am at present the Garrison Surgeon at Seringapatam; and have the charge of the first Battalion of Artillery; in consequence of which, I am superintendant of vaccination in Mysore. The latter situation is a very interesting one; and I am sure it will afford you pleasure to know, that I have very widely disseminated this invaluable blessing. Upwards of forty-two thousand were vaccinated under my superintendance in the course of last year; and in many parts parts of the peninsula, a considerable number have also undergone the disease.

The following article is extracted from the Philadelphia Medical and Physical Journal, part 1. vol. 2.

"*An account of the introduction of Vaccination into the Isles of France and Reunion.*"

This account, Dr. Barton, the editor, received from Dr. Laborde. The matter was obtained from India, and the practice became general; yet no adverse case occurred. It is supposed, that at present, there is not a single individual in either of those islands, who has not had either the small-pox or the cow-pock. A hope is therefore expressed, that the United States, quitting the inoculation of the small-pox, which only serves to perpetuate the infection of that disease, will adopt vaccination, which destroys it. Alarms have been excited in Pennsylvania, similar to those propagated in this country, concerning glandular swellings, cutaneous eruptions, and the occurrence of the small-pox after the cow-pox; but those alarms seem, in general, to have been groundless. The same disorders have been observed in those who have not undergone vaccination; and there is of course no reason to ascribe them to this cause.

Those practitioners who contend that the cow-pock occasions eruptions, or other disorders, do not seem to consider, that when they happen after vaccination, popular prejudice, together with pride, vanity, and self-interest, all concur in prompting the parent, or the patient, to attribute them all to that cause. Hence an application is commonly made to some medical man, in such cases, and they acquire notoriety; whereas, if they had been suspected to proceed from the constitution, or any other cause, they would have been concealed; lest they should bring disgrace on the family. Many cases of eruptions are now deemed important, which were once thought only to require a little physic every spring and fall.

In this metropolis, a multitude of eruptions are occasioned, in the winter, by exposing infants to severe cold; and, in the summer, by want of cleanliness. Hence we so often meet with tinea, herpes, and the itch; but still oftener, with bug-bites. These are called by various names, such as rash, eruptions, pimples, humour, scurvy, strophulus, spring eruptions, red gum, papulous eruptions, tooth rash, urticaria, nettle rash, nettle spring, erysipelas, tetters, miliary eruptions, impetigo, psora, scabies, itch, &c. &c. &c. In short, it would far exceed the limits intended tended for this letter, were I to enumerate all the names and to recapitulate all the titles, which have been conferred on this single disease. It assumes as many forms as Proteus himself; and constitutes a very great majority of all the rashes, and eruptions, that occur in this great metropolis; especially among people of the lower class. It may generally be distinguished by this characteristic; that it principally affects those parts which are most exposed.

But it is by no means confined to people in the lower class of life; on the contrary, I have very often seen it in very respectable families; and I conceive it an important duty to point out the nature of it, to prevent parents from being imposed on by despicable, unprincipled quacks; and children from being purged to death, to cure them of a bug-bite.

I knew one family, in which two children were born in the month of January, in different years; and vaccinated in February. An eruption appeared in each of them in March. Here was a coincidence, which was not considered by the medical practitioner who attended the family, though a sensible man; and vaccination was blamed, as the spring of the year frequently has been, for an eruption occasioned by vermin, then beginning to revive from their dormant state, and to venture out of their recesses. Having been desired to vaccinate one infant, whom I found almost covered with bug-bites, I requested an ingenious and experienced medical practitioner to go and see the case; and to give me his opinion concerning it. He sent me word, that it was a very infectious disorder; and, that when it appeared on one child, it generally soon afterwards appeared on others. This is no wonder, for when the bugs have bitten one child, they soon bite the rest; and the disorder spreads like wildfire.

The alarm excited by rumours of the small-pox after the cow-pock, in Pennsylvania, as well as that of eruptive and other diseases, is no more than has been excited in every other part of the world, where vaccination has been long and extensively practised; and this shews the indiscreet zeal of those advocates of vaccination, who still assert, that there have been no complaints, and no failures, but in this metropolis:

> Non tali auxilio, nec defensoribus istis,

> Tempus eget.

The truth is, that all which can justly be alleged against vaccination, is only like a spot in the sun; and not worthy to be mentioned, when we contemplate the numerous and important advantages resulting from the practice.

In

In the same Philadelphia Journal, is the very extraordinary paper, relative to vaccination, which was printed and circulated by Mr. Birch. In the copy which reached the editor, and which is there inserted, Mr. Birch had added, in writing, the case of my patient, formerly of Dorset Street, now of Wildernes Row, Salisbury Square; which is already detailed in this Journal. He has stated that I saw it, and acknowledged it to be the small-pox, which is true; and that it was full, which I deny; but he has not stated that the child never had the cow-pock in a perfect manner. This was not only my own opinion; but, according to the account of Mrs. Smith, the mother of the child, it was the opinion of every medical man who enquired into the case. After this, I could not but be surprised, that several of them, all antivaccinarians, should affirm, and affirm with an unblushing front, that this was a case of the small-pox after the cow-pox.

This transaction reminds us of one that occurred at Bath; in which a similar imposition was attempted by Mrs. Codey, an unfortunate woman of that city, who died of the small-pox. In the Bath Journal of the 19th of August, 1805, Dr. Parry informs us, that this unhappy woman, gave a false account of her child's case; and that only a few days before her death, she told various gentlemen, and others, that she had been vaccinated by Mr. Barnes of Pewsey, who assured her she would never have the small-pox; yet Mr. Barnes denies having inoculated her; and her own father and mother, and others, absolutely assert that she was inoculated by an ignorant cobler.

On Mrs. Codey's conduct, with regard to her child, Dr. Parry makes the following remarks. "This corrected narrative of Mrs. Codey, though sanctioned by the mockery of an oath, cannot therefore be true; any more than all the three accounts can, in other respects, be reconciled, or that it can be true that Mr. Edwards did not see the child during the eruption, as the woman positively asserted to us at the Vaccine Institution, and that she did see him during the eruption, as she swore before the worthy magistrate, whose name is quoted in your paper.

"On the whole, I have little doubt that this boy's complaint was the chicken pox, which has been at all periods with difficulty distinguished from the Small-pox by wiser men (with reverence be it spoken) than Mr. Edwards, and which is often preceded by violent fever, and followed by at least as deep pits as the confluent Small-pox. But then the woman swears, that the boy had had the chicken pox.

For my own part, I am very slow to trust those whom I have once detected in a falsehood; and to me it matters not, whether an untruth be told before God, or before a magistrate.

"Sir, this is a solemn cause, in which are absolutely concerned the lives and happiness of a large part of the human race, and in which any concealment of facts, or of opinions justly founded on those facts, would be as weak as the ignorance, and as criminal as the disingenousness with which we have to combat. Of this disingenousness, it is lamentable to see the extent. What shall we think of that spirit, which could induce one unfortunate woman in this city, whose case has been quoted with such triumph by the opposers of vaccinism, and, as usual, quoted only to be refuted, to rush into the presence of her Creator, with a falshood of this kind still warm upon her lips!"

Mr. Edwards of Walcot, having excited a local pustule in the arm of a child who had been vaccinated, by variolous inoculation, declared he had given him the small-pox; on which, Dr. Parry makes the following remarks.

"I positively assert that this patient, though inoculated for the small-pox, has not taken the disease; that is, to apply Mr. Edwards's test in his own words, 'as no symptoms were present, such as fever producing eruptions, I do not hesitate to say, the person was not affected with the disease.' I know that many respectable friends of Mr. Edwards, who are acquainted with this case, are ashamed of his conduct on this occasion.

"Mr. Edwards must know, that nurses, or other persons, may, by inoculation, or by contagion, frequently have local pustules, though they have previously had the inoculated or natural small-pox; and, according to his own premises above quoted, I should not admit Master Griffiths to have had the small-pox, though Mrs. Hanham, or Mrs. Battlebury, or Mr. Edwards, should, in their laudable zeal, take matter from the inoculated arm of this patient, and thus continue their malignant practice of spreading this hateful pestilence, to the destruction of half the human race.

"Let these persons go to their pillows with this reflection, and sleep on them with what comfort they may: and let them, by publicly boasting of the success of their own efforts to spread this scourge of mankind, in spite of all the efforts of the friends of humanity to expel it for ever from the face of the earth, afford a farther proof, if any were were still wanting, that insolence usually accompanies vice."

The preceding account of the introduction of vaccination into the Isle of France, is confirmed in the Asiatic Annual Register for 1804, in the following words:

"Cow-Pox.

"By late advices from the Isle of France, we have the pleasing information of the cow-pox being introduced into that colony; and that upwards of three thousand persons have been vaccinated under direction of a committee *appointed by government.*

"The inhabitants are indebted for this blessing to Captain Deglos, of the ship Phillipine; who, in March last, carried several children thither with the disease; which he kept up by successive inoculations during the voyage."

Thus this invaluable discovery is every where propagated, and every where cherished, except in its native soil; where the coldness and apathy of one class, the prejudice and ignorance of another, and the misrepresentations and falshoods of a third, have in a great measure blasted its happy fruits.

I long ago communicated the intelligence received from New South Wales, that Mr. Savage had succeeded in planting vaccination in this extensive territory; which may be considered as a fifth part of the habitable world. I now learn, that the matter, which I sent him, and which was dated October 14, 1803, was not used till May 8, 1804; so that it was, at least, seven lunar months old; and, as it was some time in collecting, part of it must have been seven calendar months. Different governments in India, despairing of success any other way, had it in contemplation to send out a number of children, in order to convey recent virus, had not that object been happily accomplished by vaccinators well charged, sent from this country.

The success of this experiment is the more remarkable, when we consider that the nearest part of New South Wales is twice as distant as the remotest part of India; and that although matter has been frequently sent from this country to India, it never succeeded there. When the last ship left New South Wales, vaccination was still practised there, as well as at Norfolk Island, and all the other settlements; and upwards of eight hundred persons have thus been protected, as far as they can be protected by human power, from the ravages of the small-pox.

The following article, also extracted from the Asiatic

Annual Register for 1804, is an additional proof of the happy progress which vaccination is still making; and of the high esteem in which it is held every where, except where it ought to be esteemed most. It is the first authentic account of its introduction into the continent of Africa.

"It is with high gratification we learn, that vaccine inoculation has been successfully introduced and established at the Cape of Good Hope, by means of a Portuguese ship arrived from Mozambic. The inhabitants universally adopted it; and made the whole of their slaves undergo the operation. So thankful, indeed, were they for the blessing, that the government of the colony permitted the vessel to prosecute her voyage, notwithstanding the strict embargo under which she had been laid."

To these important articles of intelligence, relative to the progress of vaccination in the remote parts of the world, I shall now add one from the Bibliotheque Britannique. It is an extract of a letter from Dr. Sacco, of Milan, to Professor Odier of Geneva, dated Aug. 12, 1805; of which the following is a translation.

"Having read the extract of the pamphlet of Mr. William Goldson of Portsmouth, which you have published in the Bibliotheque Britannique, I have had it greatly at heart, to put vaccination to the test of variolous inoculation, in children who have been vaccinated several years. But in the kingdom of Italy, where we already reckon more than four hundred thousand persons, who have been vaccinated, nothing is more uncommon, for these three years past, than the small-pox. Nevertheless, after many ineffectual inquiries, for the space of three months, I at length procured some fresh and select variolous matter from Cremona and Ferrara, preserved on threads and glass, taken from a child who had the confluent and perfectly unequivocal small-pox.

With this matter I inoculated, in the most careful manner, eleven children; five of whom had been vaccinated in the year 1800, five in 1801, and one in 1802. In eight of these children, the punctures produced no effect. In the three others they inflamed, suppurated, dried, and scabbed, before the 8th day, without producing any indisposition, or symptom of the small-pox. It is therefore evident, that the cow-pock is a permanent security against the small-pox: and how can this be doubted, when it is seen, that in such of our departments as have not generally adopted vaccination, the small-pox does not infect those children who have been vaccinated; although they they daily associate, in various ways, with those who labour under the small-pox, and those who are recovering from that disease?

"*I must add, that although I long since prevailed on all the clergy to offer their parishioners a reward of fifty sequins, provided they could produce a single instance, well authenticated, of the small-pox after the regular and perfect cow-pock, no one has hitherto claimed it.*

"Mr. Goldson has no less deceived himself, when he tells us of the possible degeneration of cow-pock matter, in passing from one subject to another. I have prosecuted the experiment; and accurately examined it, to the 117th generation; and hereby certify, that I have never observed the least difference between those who were vaccinated first, and those who were vaccinated last."

The Bibliotheque Britannique also contains some account of Dr. Anderson's work, relative to the progress of vaccination in India: to which is subjoined the following observation by Dr. de Carro. "When we see so great an emulation between the medical practitioners in England, and those in India, in promoting a beneficial practice, which is so free from inconvenience, and so much to be relied on, we cannot but be astonished, that it is still attacked and decried by certain physicians, in the very country which gave it birth; and that three authors have very lately published pamphlets with this design.

"Dr. Moseley's pamphlet is entitled 'Observations on the Cow-pox, or Lues Bovilla.' The very title of this work is sufficient to shew, how little the author understands, or wishes to understand, the practice of vaccination.—Dr. Squirrel has entitled his pamphlet 'Observations addressed to the public, by Benjamin Squirrel, formerly *Resident Apothecary to the Small-pox and Inoculation Hospital.*—*Hinc illæ lacrymæ!*"

Mr. Moore tell us, that "Dr. Jenner's discovery is a touchstone, intended to shew what proportion of selfishness alloys the human heart; and that it is calculated to make known, whether the scenes of misery, which medical men are compelled to witness, blunt their feelings."—He thinks the result honourable to the faculty in general; and is even inclined to believe, that the few practitioners who oppose vaccination, oppose it from a conviction, that it is not likely to be of use.—This is, indeed, a very charitable concession; and I hope a time will come, when the opponents of vaccination will give some proof that they deserve it. Hitherto appearances have been much

 against against them; and some people have doubted the sincerity of their professions, and the truth of their statements; but charity believeth all things.

One of their Commentaries now *lies* before me; in which it is pretended that several persons in the neighbourhood of Bristol, vaccinated by Dr. Jenner, have since had the small-pox, or other diseases occasioned by the cow-pox. Having sent this publication to Dr. Jenner, I am authorised to say, that the whole statement, as far as regards himself, is totally destitute of foundation. Five persons are said to have been inoculated by him; but he assures me he did not inoculate one of them.

In short, the whole statement appears to be an artful fabrication, intended to support a drooping cause. It is ascribed by the author to Mr. Sutton.

The tempting overtures made by the opponents of vaccination to the inoculators for the small-pox, and their readiness in listening to them, reminds us of two songs in the Burletta of Midas.

M. If you mean to hire for service,
Come to me you jolly dogs;
You can help to bring home harvest,
Tend the sheep, and feed the hogs.

S. I, kind Sir, accept your offer,
Farther on I may fare worse;
Zounds, I can no longer suffer
Hungry guts and empty purse.

By the harvest, we are to understand *a golden harvest*; and, by *sheep* and *hogs*, the *weak and unthinking multitude*; who, trusting to the fallacious promises of certain boasting pretenders, are led like sheep to the slaughter,

"And lick the hand just rais'd to shed their blood."

After this detail, shewing somewhat of the dark side of human nature, the following letter, which I lately received from an enlightened and disinterested physician in considerable practice, than whom, I am assured, vaccination has not a firmer friend, must afford every philanthropic mind some relief.

" *Torrington, Feb. 17th,* 1806.

" Sir,

" I should have taken an earlier opportunity of thanking you, for your polite attention in sending me the vaccinators, had I not waited the result of vaccination in my little son; who, I have the pleasure to say, has gone through that that desirable process, in a way adequate to my most sanguine wishes.

"I am truly concerned, as every man must be, who feels anxious for the improvement of medical science, to see opinions advanced, on the important subject of vaccination, with *a want of liberality*, and, I fear, in many instances, *with wilful misrepresentation, for the purpose of personal invective.*

"In this part of Devonshire, as far as my practice and information extend, vaccination has obtained a complete triumph, by a continued series of incontrovertible facts. Wishing you and your Colleages every success in the prosecution of this great and important benefit to mankind, I remain,

Sir, your much obliged,
And very humble servant,
JOHN WALDON."

Among the publications of the description alluded to by Dr. Waldon, that of Dr. Rowley stands pre-eminent. The author has since paid the debt of nature; but his book still lives; and if the numerous misrepresentations it contains be not corrected, it will still add to the incalculable mischief which it has already produced. I shall therefore continue to examine it with that freedom, which truth, and a just regard to the characters of the living require. Should I happen, in any instance, to commit an error, I shall be happy to be corrected in my turn; which can be no very difficult task, while so many of Dr. Rowley's friends, and almost all the parties concerned are still living.

In his introduction, p. IV. he says, a child of Mr. Barrell, in Phillips's Gardens, Tottenham Court Road, had the small-pox afer the cow-pock; and that the practitioner who had vaccinated the child confessed it; but declared it was an accident.—Mrs. Barrell declares that this is false.

P. VI. he says, he has introduced into his work "*a plan for prosecuting future inquiries, and preventing loss of time;*" a plan to which he most religiously adhered. It is inserted at p. 32 of his pamphlet; and may be comprised in a few words. It is as follows:

"Have the parties been inoculated for the cow-pox?"

"Have they had the small pox afterwards?"

If these questions are answered in the affirmative, we are told it is unnecessary to ask any others; that it is not material material to inquire whether the cow-pock was genuine or spurious, or even whether infection took place at all. These inquiries, he tells us, are irrelative to the question; and can only confound fools. This is the way in which he proposes, that " the most *severe investigation* into the subject should be instituted; and all idle disputation and altercation suspended." This will easily account for the long and formidable catalogue of cases, which Dr. Rowley and his Colleagues have brought forward against vaccination.

Dr. Rowley vehemently declaims against all hypotheses and conjecture; yet no man has indulged himself in hypotheses and conjectures more than he has done; it is, indeed, remarkable, and appears rather ominous, that his pamphlet begins with "*Visionary conceits, irrational projects, uncontrouled arrogance, and an obstinate perseverance in error.*"

P. 4. Dr. Rowley asserts, that the safety and certainty of small-pox inoculation is established by the successful experience of a hundred years. This is not correct. The inoculation of the small-pox, it is well known, is neither safe, nor certain; and it has not been regularly practised in this kingdom a hundred years.

He tell us, that this inoculation had arrived at the utmost degree of perfection; and that it was next to impossible to lose an inoculated patient out of many thousands. He says, the certainty of safety and securely preventing the small-pox "had been infallibly proved by *millions*, nay *myriads*, of instances."—Here he represents ten thousand to be more than a million. This is one among the many instances of ignorance in the opponents of vaccination, who call others ignorant and illiterate; and treat them with contempt.

He says, the "*safety* and *certainty* of variolous inoculation are proved, beyond the possibility of *doubt*, cavil, or disputation."—This is refuted by the experience of every civilized country; every day, and every hour.

He says, a new disease from the brute creation, called the cow-pox, has been "ushered into the world precipitately and vehemently."—It was, however, in the world long ago; and, in the opinion of the most learned and enlightened part of the community, has not been ushered into medical practice with half the zeal which its importance demands.

Dr. R. says, the practice of the inoculation of the small-pox is safe, mild, and certain. Mr. Moore, however, in his Reply

Reply to the Antivaccinists, is bold enough to controvert this position. He says, p. 64, "the improved method of treating the small-pox renders it less destructive now than it formerly was; and inoculation *commonly* makes it safe to the person inoculated. A part, however, of those who are inoculated die. This is admitted by all *eminent* and *candid* physicians. The proportion of deaths it is difficult to ascertain; no practitioner, *for obvious reasons,* being willing to acknowledge *the whole truth* on this delicate point. All writers, however, agree in this, that *the confluent small-pox* is sometimes produced by inoculation. They likewise mention *epileptic fits* as a frequent occurrence; *and there is no man, whose testimony is worth listening to, who can dare assert, that the confluent small-pox, and epileptic fits, are not very alarming circumstances.*

"As these are *very common,* some, and especially the weakly, must sink under them. This is so well known, *that no physician, or surgeon, who had any regard for truth, or for his own character, ever pretended that the operation was void of danger.* The number of deaths has been estimated at one in a hundred, or one in two hundred; and there is no reason to suspect, that practitioners have exaggerated their ill success."

Mr. Blair, in his able publication, entitled The Vaccine Contest, observes, that during the preceding year, prejudices against vaccine inoculation were excited with great industry, by the different opponents of vaccination; some of whose names and deeds will probably be long remembered by the public, with feelings of honest indignation. He tells us, that the falsehoods and misrepresentations which were propagated at times, had such an effect, as to put a total stop to vaccination at the Bloomsbury Dispensary for upwards of three months. In consequence of this unfortunate occurrence, Mr. Blair thought it his duty to read Dr. Rowley's vaunting pamphlet; of the tendency of which he was in some measure previously informed, by means of puffing handbills, newspaper paragraphs, and disgraceful handbills upon all the dead walls of London.

Mr. Blair is of opinion, that he has never read a publication so unfair, so insidious, so deceitful, so mischievous, so full of invective, so full of falsehood, so inconsistent, or so repugnant to decency, as that of Dr. Rowley. Hence he conceived the idea "of turning against an implacable adversary, the murderous weapons which he himself had provided for another purpose." He observes, serves, that although it is an ungrateful task, it would not be lost time, to extract the essence of Dr. Rowley's extraordinary performance; and to shew its genuine deformity. "The real character and motives of an opponent, *who is so entirely devoid of justice and decorum,* cannot be better discovered, than by dissecting, analysing, and exposing to public view, what may be called the vitals and sinews, the internal springs, the peculiar features and tone of his composition: and if it should be found, that his character and motives are far from being pure, except in his own eyes, it may be questioned whether his pretended *truths* are unexceptionable, or his alleged *facts* such as honesty demands."

Mr. Blair next observes, that in undertaking this task, he cannot justly be suspected of acting from personal pique or resentment, in consequence of insults received by him, in common with many of his medical friends; since he is "*one of the few practitioners, whom Dr. Rowley has never offended.*"—He acknowledges, however, that the *praise* of such an author is worse than his *censure*.

He tells us, in a postscript, while his pamphlet was in the press, he was informed, that Dr. Rowley had been summoned to another, and more awful tribunal, than that of man. But, he adds, as what he has written in answer to him is of equal concern to the public, whether the great champion of antivaccination is living or not; and as the errors which were so industriously propagated by him, are still kept alive by others, and not likely to be soon eradicated, he could not refrain from publishing, what he had conscientiously written for the good of mankind.

At the conclusion of his work, Mr. Blair introduces some remarks on the comparative advantages of variolous and vaccine inoculation, under the title of Hints to Parents. He there describes the havoc of the small-pox, and the miseries entailed on those who survive the ravages of that dreadful disease. He then subjoins the following observations, which deserve to be read with great attention.—"Should you think to prevent all these distressing consequences, by inoculating your children for the small-pox, it is to be remembered, that the same kind of disorders may follow inoculation; although the danger is thereby much lessened. There is, however, one distressing consequence, produced by inoculation, which you may not always consider. Your neighbours may thus be exposed to a distemper, which did not before exist among them. The infant whom you inoculate may escape with life; life; but the children of others may catch the small-pox from him and perish. Let me notice the danger of your own child's situation. One in two hundred, and often a still greater number, will die of the small-pox, when produced by inoculation; and the circumstance of your having voluntarily exposed your infant to a fatal disease, will ever be a bitter subject of reflection."

He expresses his opinion, that Dr. Rowley's *matters of fact*, as he calls them, and his *Antivaccinarian Society*, are a disgrace to the medical profession; that he has impeached the moral characters of those, who are far his superiors in reputation and virtue; that his pamphlet is a gross libel on the characters of men of the first celebrity, and merits no other title, than that of being a most scandalous vehicle of falsehood.

Mr. Blair also notices the degree of estimation in which Dr. Rowley was held among the respectable and honourable part of the faculty. He notices his puffs, his handbills; his humiliating attentions to self-dubbed doctors and apothecaries, and the feasts with which he was continually treating them, both in town and country, by way of a bribe, in order to prevail on them to consult him, and to countenance his artful and self-interested plans.

Ambubaiarum collegia, pharmacopolæ,

Mendici, mimæ, balatrones, — hoc genus omne

Moestum ac sollicitum est cantoris morte Tigelli,

Quippe *benignus* erat.

So said Horace long ago; and had he been living now, he could not have expressed himself in more appropriate terms. It is not a regard for the character of *Dr. Rowley*, but *for their own*, which must stand or fall with his, and a regret for the gratifications they have lost, that has extorted such high-flown panegyrics from the pens of his surviving colleagues. Compassion to their present abject state, would induce us to be silent, and not to trample on fallen enemies; "but," to use the words of a celebrated political* writer, "when facts are so daringly challenged, compassion would cease to be a virtue."

I am, &c.

JOHN RING.

*New Street, Hanover Square,*
*May* 14, 1806.

* Junius.

